# IgG4-sclerosing cholangitis masquerading as cholangiocarcinoma: a case report of an unresolved preoperative diagnosis

**DOI:** 10.3389/fimmu.2026.1713456

**Published:** 2026-04-02

**Authors:** Ying-Ao Liu, Xiang Gao, Wei Gao, Zeliang Hu, Fangzhou Wang, Yuanxu Qu, Yamin Zheng

**Affiliations:** 1 Department of General Surgery, Xuanwu Hospital, Capital Medical University, Beijing, China; 2 Department of Pathology, Xuanwu Hospital, Capital Medical University, Beijing, China

**Keywords:** cholangiocarcinoma, IgG4, IgG4-related disease, IgG4-related sclerosing cholangitis, pancytopenia

## Abstract

**Background:**

Immunoglobulin G4-related disease (IgG4-RD) is an immune-mediated chronic fibroinflammatory condition that can affect multiple organ systems. IgG4-related sclerosing cholangitis (IgG4-SC) is its manifestation involving the biliary tract. Due to atypical clinical presentations in some cases and insufficient awareness among clinicians, IgG4-SC is frequently misdiagnosed as cholangiocarcinoma, and multiple instances of inappropriate treatment as a result have been documented.

**Case presentation:**

Here we report a case of IgG4-SC that presented diagnostic challenges preoperatively and was followed by rare pancytopenia after surgery. The patient was a 50-year-old man who sought medical attention due to elevated transaminases for eight months, without obvious clinical symptoms. Among serum tumor markers, the level of protein induced by vitamin K absence/antagonist-II (PIVKA-II) was elevated. Examinations including magnetic resonance cholangiopancreatography (MRCP), contrast-enhanced abdominal computed tomography (CT), and positron emission tomography-computed tomography (PET-CT) all suggested malignant stricture at the hepatic hilum. A needle biopsy indicated dysplastic changes but was inconclusive for cholangiocarcinoma. Although IgG4-SC was considered, multiple serum IgG4 measurements remained within the normal range. With an initial clinical diagnosis of cholangiocarcinoma, the patient underwent surgical resection. Intraoperative frozen section analysis indicated an inflammatory process. Postoperatively, the patient developed pancytopenia that responded poorly to conventional supportive treatment. The condition was ultimately diagnosed as IgG4-SC based on postoperative histopathology. Corticosteroid therapy led to the normalization of the patient’s blood counts, transaminases, and bilirubin levels.

**Conclusion:**

IgG4-SC can present with a spectrum of atypical features, such as isolated biliary strictures, absence of characteristic symptoms, normal IgG4 levels, and imaging findings resembling cholangiocarcinoma. Therefore, in cases clinically suspicious for cholangiocarcinoma, differentiating IgG4-SC warrants serious consideration. Every effort should be made to complete preoperative biopsy and histopathological assessment. For cases where differentiation remains difficult despite comprehensive evaluation, surgical intervention with intraoperative frozen section biopsy is necessary to establish a definitive diagnosis and avoid delayed treatment. Furthermore, this case suggests that IgG4-SC may also involve the hematopoietic system, manifesting as pancytopenia, which can respond effectively to corticosteroid therapy.

## 1 Introduction

Immunoglobulin G4-related disease (IgG4-RD) is a systemic immune-mediated condition characterized by elevated serum IgG4 levels and dense infiltration of IgG4-positive plasma cells in affected tissues (1, 2) This disease triggers chronic immune activation, leading to tissue damage dominated by fibrosis (3, 4). IgG4-RD was initially identified in cases of autoimmune pancreatitis and, with growing research, has been recognized as a distinct systemic disorder capable of involving multiple organs (5). Commonly affected sites include the pancreas, bile ducts, gallbladder, lungs, and kidneys, with over 75% of patients exhibiting simultaneous involvement of two or more organs (6–9). When the biliary system is affected, it manifests as IgG4-related sclerosing cholangitis (IgG4-SC).

Approximately 90% of IgG4-SC patients exhibit elevated serum IgG4 levels, which has become an important supportive criterion for diagnosis (10, 11). However, IgG4-SC closely resembles cholangiocarcinoma in both clinical presentation and imaging features. Coupled with the fact that IgG4-SC has only recently been recognized and clinical awareness remains limited, there is often a lack of alertness regarding its differential diagnosis. In recent years, there have been repeated reports of cases misdiagnosed as cholangiocarcinoma and undergoing surgery, with the correct diagnosis only established postoperatively through pathological examination (12–17). It is noteworthy that the treatment strategies for these two conditions differ completely: surgical resection is the mainstay for cholangiocarcinoma, whereas glucocorticoid therapy is first-line for IgG4-SC (18). Therefore, improving the ability to differentiate IgG4-SC from cholangiocarcinoma is of significant clinical importance.

This article presents an exceptionally challenging case of IgG4-SC that was ultimately confirmed through surgery and pathology. Drawing insights from this particular case, we aim to discuss key points in the diagnosis and treatment decision-making between IgG4-SC and cholangiocarcinoma, so as to enhance clinicians’ ability to recognize and manage atypical presentations of IgG4-SC.

## 2 Case presentation

**Figure 1:**
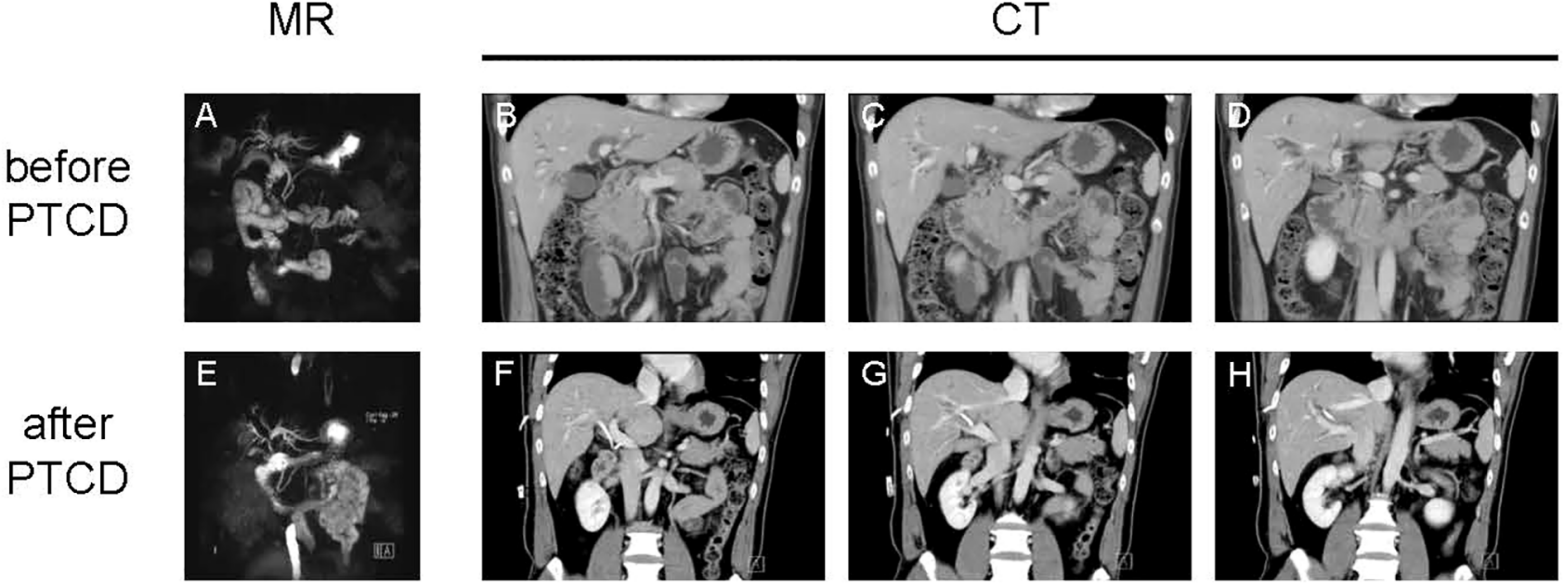
Radiological evaluation of the patient before and after percutaneous transhepatic cholangial drainage (PTCD). **(A)** Reconstructed MRCP image prior to PTCD demonstrating high biliary obstruction. **(B–D)** Representative contrast-enhanced CT images before PTCD showing a slightly low-density mass at the hepatic hilum with slightly thickened bile duct walls and lumen narrowing (red arrowheads indicate the enhanced mass and thickened walls). **(E)** Reconstructed MRCP image after PTCD showing the drainage tube and alleviated intrahepatic duct dilation. **(F–H)** Post-PTCD contrast-enhanced CT images demonstrating the stable, persistently enhanced mass at the hepatic hilum.

The patient provided written informed consent for the publication of this case report. In November 2024, a 50-year-old male patient was admitted to our hospital with the chief complaints of “elevated transaminases for over 8 months, and discovered hilar bile duct occupancy for 2 months”. In March 2024, the patient was found to have mildly elevated transaminases (ALT approximately 80 U/L) during a routine physical examination, without obvious clinical symptoms such as fever, gastrointestinal issues, or jaundice. The patient received hepatoprotective therapy at an external hospital, and over the following 8 months, transaminase levels fluctuated and increased, yet the patient remained asymptomatic. In September 2024, magnetic resonance cholangiopancreatography (MRCP) and contrast-enhanced abdominal computed tomography (CT) scans revealed intrahepatic bile duct dilation, hilar bile duct stenosis, and a space-occupying lesion (**Figure 1**). Positron emission tomography-computed tomography (PET-CT) imaging showed high metabolism in the hilar lesion, suggesting a high probability of malignancy. In November of the same year, the patient underwent spyglass-assisted endoscopic retrograde cholangiopancreatography (ERCP) with biopsy at another hospital; pathology showed no definitive evidence of malignancy. For further diagnosis and treatment, the patient was transferred to our department and admitted with a diagnosis of “hilar occupying lesion”.

In November 2024, an abdominal CT at our hospital revealed a slightly low-density hilar mass (approximately 46 HU) with slight thickening of the bile duct wall and lumen narrowing. Following contrast administration, the mass and the thickened bile duct wall exhibited moderate enhancement across the arterial, venous, and delayed phases (64, 95, 95 HU).

Laboratory tests showed normal levels of CEA, AFP, and CA19-9, but protein induced by vitamin K absence/antagonist-II (PIVKA-II) was significantly elevated (680 mAU/mL). Dynamic monitoring of cholestasis markers before drainage revealed elevated alkaline phosphatase (ALP) at 355 IU/L, gamma-glutamyl transferase (GGT) at 466 IU/L, and total bilirubin (TBIL) peaking at 34.00 μmol/L. The patient previously underwent an ERCP biopsy at another hospital; however, the pathology only captured broken columnar epithelial mucosa with mild atypical hyperplasia. Because the biopsy failed to retrieve deeper tissue layers, it was diagnostically inconclusive. Repeated serum IgG4 levels were within normal limits. Based on the elevated PIVKA-II, dysplastic changes, and highly suspicious imaging, a primary diagnosis of cholangiocarcinoma was considered. Contrast-enhanced MRI was not performed as the combined findings of contrast CT, MRCP, and PET-CT were already highly indicative of malignancy. A pre-operative liver biopsy was also avoided to prevent needle-tract seeding and because typical IgG4-SC features are rarely captured in superficial or non-targeted liver biopsies. Short-term steroid trials were strictly withheld due to the severe risk of promoting tumor progression if the lesion was indeed malignant.

After percutaneous transhepatic cholangial drainage (PTCD) in December 2024, the patient’s cholestasis partially resolved (TBIL dropped to 8.36 μmol/L, ALP to 277 IU/L, and GGT to 179 IU/L by Dec 31). Following portal vein embolization (PVE) to induce liver hypertrophy, the patient underwent right hepatectomy and hepaticojejunostomy on January 7, 2025. Intraoperative frozen sections revealed extensive lymphocyte and plasma cell infiltration but no definite malignant cells. Notably, frozen sections are limited as they cannot perform IgG4 immunostaining; thus, only a provisional “inflammatory process” could be suggested intraoperatively.

Postoperatively, the patient developed severe, progressive pancytopenia. By January 20, his white blood cell (WBC) count dropped to $1.89 \times 10^9/L$. Granulocyte colony-stimulating factor (G-CSF) was administered, which transiently improved WBCs, but hemoglobin fell to 66 g/L (Jan 26), and platelets plummeted to a nadir of $12 \times 10^9/L$ (Jan 29). To rule out drug-induced bone marrow suppression, a multidisciplinary consultation was held on January 24. Suspect medications (ursodeoxycholic acid, magnesium isoglycyrrhizinate, and cefminox) were immediately discontinued. However, the patient’s blood counts did not recover post-cessation, and a hematology consultation ruled out primary hematological malignancies.

**Figure 2:**
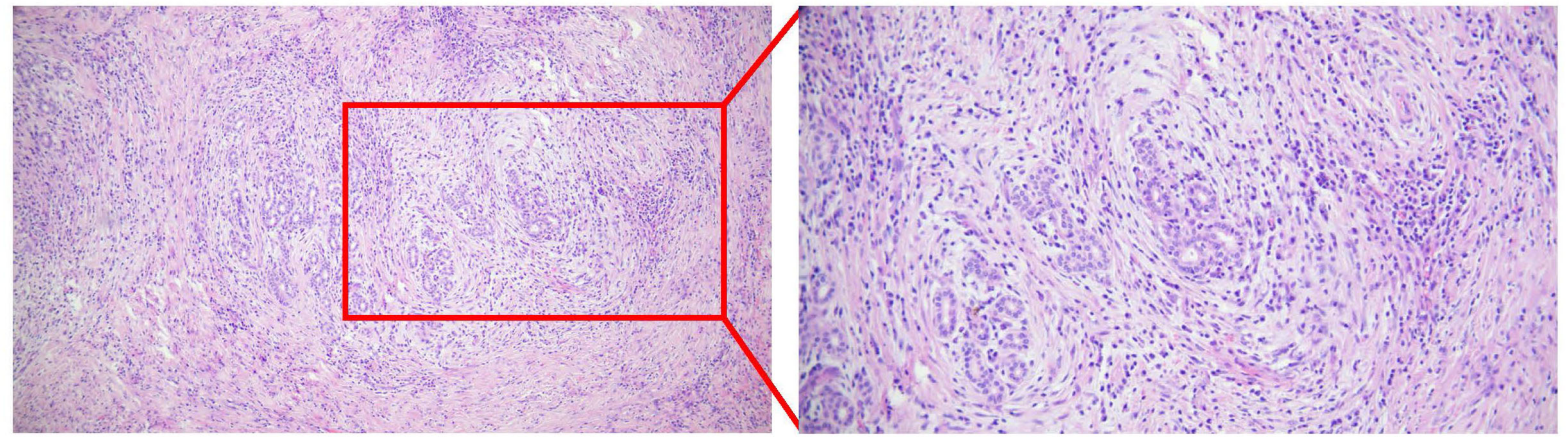
Postoperative histological features of the resected hilar lesion. A Photomicrograph of the postoperative formalin-fixed paraffin-embedded (FFPE) tissue section stained with hematoxylin and eosin (H&E), demonstrating dense lymphoplasmacytic infiltration, storiform fibrosis, and obliterative phlebitis, confirming the diagnosis of IgG4-related sclerosing cholangitis (IgG4-SC). (Note: Digital microphotographs of the IgG4 immunohistochemical staining were not captured prior to slide archiving, but the official pathology report confirmed up to 50 IgG4+ plasma cells per high-power field, with an IgG4/IgG ratio of approximately 30%).

Postoperative formalin-fixed paraffin-embedded (FFPE) pathology finally confirmed IgG4-SC, demonstrating dense lymphoplasmacytic infiltration, storiform fibrosis, and obliterative phlebitis (**Figure 2**). Immunohistochemistry confirmed up to 50 IgG4-positive plasma cells per high-power field (IgG4/IgG ratio ~30%). Corticosteroid therapy was promptly initiated, after which the patient’s blood counts and liver enzymes successfully normalized.

## 3 Discussion

Apart from the pancreas, the biliary system is the most frequently involved organ in IgG4-RD, specifically manifesting as IgG4-SC (19). Typical IgG4-SC often presents with obstructive jaundice, diffuse or segmental thickening of the bile duct wall, frequently accompanied by elevated serum IgG4 levels, and a good response to glucocorticoid therapy (20). Due to its high clinical and radiological similarity to cholangiocarcinoma, misdiagnosis has been frequently reported, with most patients only being definitively diagnosed through postoperative pathology. However, even when clinicians maintain a high degree of vigilance and actively pursue differential diagnosis, some cases of IgG4-SC present with highly atypical clinical features, making preoperative differentiation extremely challenging.

This article reports a particularly unique case of IgG4-SC that exhibited atypical characteristics in terms of clinical symptoms, lesion location, imaging findings, tumor markers, IgG4 levels, and preoperative pathology. Although differential diagnosis from cholangiocarcinoma was emphasized throughout the diagnostic process, comprehensive evaluations consistently pointed toward a malignant diagnosis. The patient was ultimately diagnosed with IgG4-SC only after surgery and postoperative pathological examination. Postoperatively, the patient developed rare pancytopenia, which responded poorly to conventional supportive treatment but improved rapidly with glucocorticoid therapy. This may represent a systemic immune response involving the hematopoietic system triggered by the surgery.

Both cholangiocarcinoma and IgG4-SC can present with non-specific symptoms such as obstructive jaundice, abdominal pain, pruritus, weight loss, and fever (21). Notably, approximately 25% of IgG4-SC patients exhibit only abnormal liver function tests without typical clinical symptoms (22, 23). Imaging is a crucial tool for the initial evaluation of biliary diseases. Both conditions can show signs such as biliary strictures, wall thickening, and upstream biliary dilation (24). Key imaging differentiating features include: in IgG4-SC, the bile duct wall shows uniform thickening in both stenotic and non-stenotic segments with clear boundaries and preserved lumen morphology; whereas cholangiocarcinoma typically exhibits irregular thickening with blurred margins. IgG4-SC predominantly affects the distal bile duct and is often associated with multi-organ involvement (e.g., pancreas, salivary glands, retroperitoneum), while cholangiocarcinoma is more common in the hepatic hilum, with consistent locations of stricture and thickening, potentially accompanied by lumen obliteration (25, 26). Nevertheless, imaging examinations only play an auxiliary role in diagnosis, and even experienced radiologists often find it difficult to make accurate judgements. This case involved a solitary hilar lesion, which is relatively uncommon in IgG4-SC, and showed significant enhancement in the venous and delayed phases of contrast scans. Multiple imaging evaluations highly suggested malignancy, highlighting the diagnostic challenges of atypical IgG4-SC.

In addition to CT and MRI, endoscopic ultrasound (EUS) and intraductal ultrasound (IDUS) can clearly visualize bile duct wall structure, thickness, and lumen morphology, providing significant value in differentiating IgG4-SC from cholangiocarcinoma (27). Characteristic features include concentric symmetric wall thickening, smooth margins, distinct layers, and homogeneous internal echogenicity (28). Studies indicate that EUS can differentiate IgG4-SC from cholangiocarcinoma with a sensitivity and specificity of up to 86% and 95%, respectively (29, 30). IDUS findings showing a bile duct wall thickness exceeding 0.8 mm in non-stenotic areas are highly suggestive of IgG4-SC (31, 32).

Serum tumor markers also play an important role in differential diagnosis. Most cholangiocarcinoma patients show elevated CA19–9 and CEA, while a minority may have elevated PIVKA-II (33). Although tumor markers have limited specificity, they still provide valuable reference. In this case, consistently normal CA19–9 and CEA levels somewhat argued against cholangiocarcinoma; however, significantly elevated PIVKA-II suggested possible malignancy, adding diagnostic confusion.

Serum IgG4 level is a key criterion for diagnosing IgG4-SC, elevated in approximately 90% of patients, particularly during active disease or with multi-organ involvement (10, 11). The Japan Biliary Association recommends suspecting IgG4-SC when IgG4 ≥135 mg/dL (34). However, it is important to note that about 10%–20% of cholangiocarcinoma patients may also show mildly elevated IgG4, and some IgG4-SC patients, like the one in this case, can have serum IgG4 levels within the normal range. Therefore, indices such as the IgG4/IgG ratio and IgG4/IgG RNA ratio have been proposed to improve diagnostic accuracy (35).

When non-invasive tests are inconclusive, pathological biopsy becomes the gold standard (36). Typical histological features of IgG4-SC include dense lymphoplasmacytic infiltration, storiform fibrosis, obliterative phlebitis, and eosinophilic infiltration. Immunohistochemistry requires >10 IgG4^+^ plasma cells per high-power field (HPF) and an IgG4^+^/IgG^+^ cell ratio >40% (18, 35). Common biopsy methods include obtaining tissue via PTCD or ERCP. Since the biliary epithelium in IgG4-SC patients is often normal, biopsy samples must include the submucosa or deeper layers of the bile duct, which is challenging with endoscopic forceps. As IgG4-SC can affect both extrahepatic and small intrahepatic bile ducts, liver biopsy might yield typical IgG4-SC pathology. However, literature reports indicate that only about 25% of IgG4-SC cases show typical small bile duct involvement; most only display portal inflammatory cell infiltration, resulting in a low positive rate for typical IgG4-SC pathology via liver biopsy (22). This might explain why biopsies via ERCP and PTCD in this case failed to provide a definitive diagnosis. Furthermore, some reports suggest that endoscopic ultrasound-guided fine-needle aspiration (EUS-FNA) has unique advantages in diagnosing IgG4-SC and perhaps should be more actively promoted in clinical practice (37).

If IgG4-SC cannot be ruled out after comprehensive evaluation but malignancy remains a possibility, surgical intervention offers both diagnostic and therapeutic value. On one hand, although IgG4-SC is a chronic inflammatory condition, one long-term follow-up study suggested it might be a paraneoplastic syndrome with a higher long-term risk of carcinogenesis than the general population (38–42), and cases of co-occurrence with cholangiocarcinoma have been reported (15, 43, 44). On the other hand, if the patient actually has cholangiocarcinoma, delayed surgery may miss the chance for curative resection, while blind trial glucocorticoid therapy carries the risk of promoting tumor progression. Therefore, surgery after multidisciplinary assessment is a reasonable choice, but the extent of resection should be adjusted promptly based on intraoperative frozen section pathology to avoid excessive removal.

The diagnosis of IgG4-SC is notoriously challenging when it mimics cholangiocarcinoma. According to the 2020 revision of the clinical diagnostic criteria for IgG4-SC by the Japan Biliary Association (Nakazawa T et al., 2021), diagnosis relies on a combination of biliary imaging, serum IgG4 levels, extra-biliary organ involvement, and histology. Our patient represented a diagnostic dilemma preoperatively: he presented with a localized hilar stricture without characteristic diffuse wall thickening, normal serum IgG4 levels, and no other organ involvement. Therefore, preoperative diagnosis was virtually impossible under standard criteria, and the definitive diagnosis was only met postoperatively through definitive histology (Criterion IIIa).

This case highlights the limitations of standard biopsy techniques. Endoscopic forceps biopsies (via ERCP) often only obtain superficial mucosa, which explains the inconclusive preoperative pathology in our patient. Therefore, Endoscopic Ultrasound-Guided Fine-Needle Aspiration (EUS-FNA) should be emphasized in clinical practice. EUS-FNA allows for targeted sampling of the submucosa and deeper layers of the bile duct wall where IgG4-positive plasma cells primarily reside, significantly enhancing preoperative diagnostic accuracy for IgG4-SC. Furthermore, when surgery is inevitable, intraoperative frozen section analysis plays a crucial role as a safety net. Although frozen sections cannot perform IgG4 immunostaining to definitively confirm IgG4-SC, they can identify dense lymphoplasmacytic infiltration and exclude typical adenocarcinoma, preventing the patient from undergoing excessively radical resections.

The most remarkable feature of this case was the severe postoperative pancytopenia. Drug-induced bone marrow suppression was systematically ruled out as blood counts failed to recover after discontinuing suspect medications. Recently, Liu Z et al. reported that IgG4-RD can be accompanied by primary myelofibrosis, indicating that the disease can directly involve the hematopoietic system. The sudden onset of pancytopenia postoperatively in our patient might represent a systemic immune storm or exacerbation of bone marrow involvement triggered by the surgical trauma. The rapid and robust recovery of all three blood cell lineages following systemic corticosteroid administration strongly supports this immune-mediated pathogenesis, expanding our understanding of the systemic manifestations of IgG4-SC.

## 4 Conclusion

Based on this challenging case, several specific clinical insights can be drawn:

1. In patients presenting with suspected cholangiocarcinoma—even if serum IgG4 levels are normal and clinical symptoms are atypical—IgG4-SC must be kept in the differential diagnosis, especially when dysplastic changes are found without overt malignancy.
2. Preoperative EUS-FNA is highly recommended over standard superficial biopsy to obtain deeper submucosal tissue for accurate IgG4 staining. When preoperative confirmation fails, surgery combined with intraoperative frozen section analysis is the critical final defense to avoid both delayed cancer treatment and unnecessarily radical resections.
3. IgG4-SC can manifest with rare, severe systemic immune reactions such as postoperative pancytopenia. When drug-induced causes are ruled out, clinicians should recognize this as a potential systemic manifestation of IgG4-RD, which can be effectively salvaged with corticosteroid therapy.

## Data Availability

The original contributions presented in the study are included in the article/supplementary material. Further inquiries can be directed to the corresponding author.
